# A Case of Refractory Polyarticular Juvenile Idiopathic Arthritis Post-hematopoietic Stem Cell Transplantation Successfully Treated With Ruxolitinib

**DOI:** 10.7759/cureus.104044

**Published:** 2026-02-22

**Authors:** Yuta Sonehara, Yuta Maruyama, Kazuo Sakashita, Yozo Nakazawa

**Affiliations:** 1 Department of Pediatrics, Nagano Red Cross Hospital, Nagano, JPN; 2 Department of Pediatrics, Shinshu University School of Medicine, Matsumoto, JPN; 3 Department of Hemato-Oncology, Nagano Prefectural Children’s Hospital, Azumino, JPN

**Keywords:** allogeneic hematopoietic stem cell transplantation (hsct), graft-versus-host disease (gvhd), janus kinase inhibitor, juvenile idiopathic arthritis (jia), ruxolitinib

## Abstract

Allogeneic hematopoietic stem cell transplantation (allo-HSCT) may trigger secondary autoimmune diseases, with autoimmune arthritis representing a rare yet challenging complication.

Here, we report a six-year-old male who developed polyarticular juvenile idiopathic arthritis (JIA) three years after allo-HSCT for lymphoma. Despite resistance to multiple biologic disease-modifying anti-rheumatic drugs (bDMARDs), including adalimumab and tocilizumab, treatment with the Janus kinase 1/2 inhibitor ruxolitinib achieved clinical remission sustained for over two years without significant adverse events or lymphoma relapse.

Ruxolitinib may represent a viable therapeutic option for refractory secondary JIA post-allo-HSCT, particularly when conventional bDMARDs fail.

## Introduction

Allogeneic hematopoietic stem cell transplantation (allo-HSCT) provides curative treatment for various malignant and non-malignant diseases. However, immune reconstitution post-transplantation may paradoxically trigger secondary autoimmune diseases (ADs), with a reported incidence of approximately 5-6.6% [1]. The most common manifestations are hematological disorders such as immune thrombocytopenia, autoimmune hemolytic anemia, and Evans syndrome. Among secondary ADs, autoimmune arthritis, including rheumatoid arthritis (RA) and juvenile idiopathic arthritis (JIA), represents a rare but challenging complication [1,2]. Distinguishing de novo autoimmune arthritis from chronic graft-versus-host disease (GVHD)-related arthropathy, which typically presents as joint contractures, is a well-recognized diagnostic challenge [2].

Herein, we report a patient with polyarticular JIA after allo-HSCT who remained refractory to multiple biologic disease-modifying anti-rheumatic drugs (bDMARDs), including a tumor necrosis factor (TNF) inhibitor and an interleukin-6 (IL-6) receptor inhibitor. The patient responded successfully to the Janus kinase (JAK) inhibitor ruxolitinib (Ruxo), suggesting a potential therapeutic approach for this rare and treatment-resistant condition.

## Case presentation

A six-year-old male with refractory anaplastic large cell lymphoma and no personal or family history of AD underwent allogeneic bone marrow transplantation from his human leukocyte antigen (HLA)-mismatched (2/8 allele, graft-versus-host direction) mother. The preconditioning regimen consisted of fludarabine, cyclophosphamide, and 8 Gy total body irradiation. GVHD prophylaxis was administered with tacrolimus and short-term methotrexate (MTX).

Post-transplantation, he developed acute GVHD with skin (grade 1) and severe gastrointestinal (grade 4) involvement, which was treated with methylprednisolone, mycophenolate mofetil, azathioprine, and rituximab. Two years post-HSCT, he presented with muscle weakness and was diagnosed with polymyositis with negative myositis-related autoantibodies, requiring increased corticosteroid dosage and rituximab.

**Figure 1:**
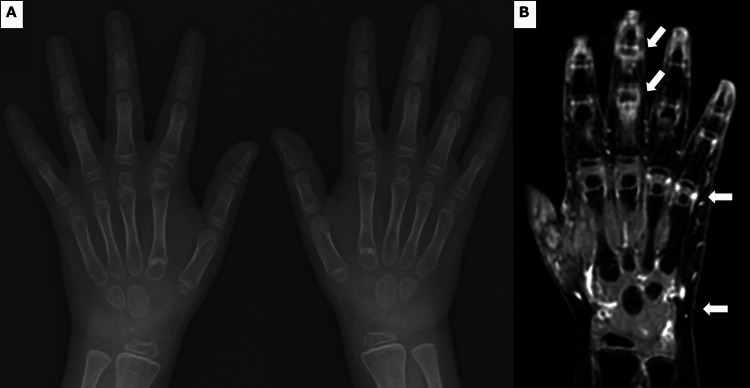
Imaging findings of the hands and wrists at the onset of juvenile idiopathic arthritis (A) Anteroposterior radiograph of both hands showing no evident bone erosions or joint space narrowing. (B) Contrast-enhanced T1-weighted magnetic resonance imaging of the wrists and fingers revealing active synovitis and joint effusion. White arrows indicate active synovitis and joint effusion.

At age 12 (three years post-HSCT), while receiving maintenance therapy with prednisolone (PSL) (0.2 mg/kg/day) and tacrolimus (trough level approximately 2 ng/mL), he developed symmetrical polyarthritis with pain, swelling, and morning stiffness, predominantly affecting the metacarpophalangeal and proximal interphalangeal joints of both hands, wrists, and knees. Body weight was 20 kg. Although symptoms temporarily improved with increased PSL dosage of 10 mg/day (0.5 mg/kg/day), he became steroid-dependent and was referred to our department. Laboratory findings revealed an elevated C-reactive protein of 2.99 mg/dL (reference range: <0.3 mg/dL) and matrix metalloproteinase-3 (MMP-3) of 141 ng/mL (adult reference range: 36.9-121 ng/mL). He tested positive for antinuclear antibody (ANA) (1:160, homogeneous pattern, reference range: <1:40) but negative for rheumatoid factor and anti-cyclic citrullinated peptide antibody. Although specific autoantibodies such as anti-double-stranded DNA (anti-dsDNA) and anti-Smith (anti-Sm) were not measured, the patient lacked other clinical features of systemic lupus erythematosus. Hand radiographs showed no bone erosions (Figure 1A); however, contrast-enhanced magnetic resonance imaging confirmed synovitis in wrist and finger joints (Figure 1B).

**Figure 2:**
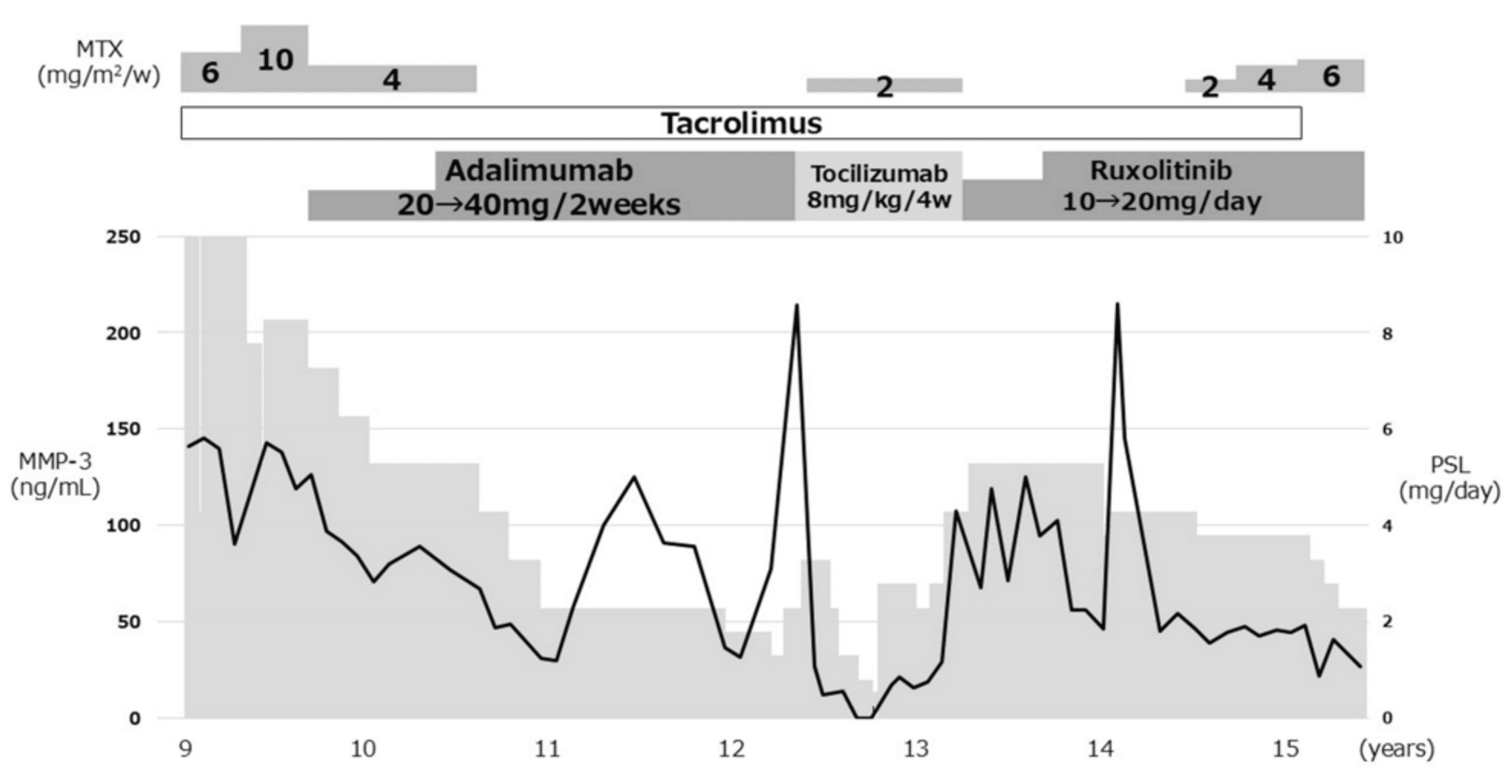
Clinical course and treatment response The graph illustrates the patient's clinical progress following the onset of polyarticular juvenile idiopathic arthritis. The transition of medical treatments is shown alongside the changes in inflammatory markers. Clinical improvement is reflected by the normalization of matrix metalloproteinase-3 levels. Abbreviations: MMP-3, matrix metalloproteinase-3; MTX, methotrexate; PSL, prednisolone.

Based on these findings, he was diagnosed with rheumatoid factor-negative polyarticular JIA according to International League of Associations for Rheumatology (ILAR) criteria. At the time of diagnosis, the clinical Juvenile Arthritis Disease Activity Score in 27 joints (cJADAS-27) was 30, indicating high disease activity. Despite treatment with corticosteroids, MTX, and tacrolimus, disease activity remained high. MTX was discontinued due to adverse effects. Sequential bDMARD therapy was administered; although initially effective, both the TNF inhibitor adalimumab (ADA) and IL-6 receptor inhibitor tocilizumab (TCZ) demonstrated secondary inefficacy within one year, failing to maintain disease control. The JAK 1/2 inhibitor Ruxo was initiated at 10 mg/day and increased to 20 mg/day for better disease control. This produced prompt and significant improvement in joint symptoms and normalization of inflammatory markers, allowing successful PSL tapering from 10 mg/day to 2 mg/day (Figure 2).

Although a transient increase in MMP-3 levels was observed after age 14, the patient had no active joint symptoms or extra-articular manifestations, and it resolved spontaneously without any therapeutic intervention or clinical symptoms. No significant adverse events, such as cytopenia or severe infections, occurred during Ruxo treatment. No evidence of lymphoma relapse was observed throughout this period. At last follow-up (aged 15 years, weighing 30 kg), the patient maintained clinical remission for over two years (JADAS-27 score of 0, indicating inactive disease) on a minimal PSL dose of 0.067 mg/kg/day (2 mg/day) without secondary inefficacy.

## Discussion

We report a case of bDMARD-refractory polyarticular JIA that developed after allo-HSCT and was successfully treated with the JAK inhibitor Ruxo. This case demonstrates a potential therapeutic approach for a rare and treatment-resistant complication.

Although secondary ADs are recognized complications of allo-HSCT, autoimmune arthritis such as RA and JIA is particularly rare compared to hematological manifestations [1,2]. As summarized in Table 1, many reported cases of post-HSCT arthritis involve a high prevalence of autoantibodies or coexistence of other autoimmune conditions like Evans syndrome [1-5].

**Table 1:** Summary of Reported Cases of Rheumatoid Arthritis or Juvenile idiopathic Arthritis After HSCT Abbreviations: ADA, adalimumab; ADs, autoimmune diseases; ALL, acute lymphoblastic leukemia; AML, acute myelogenous leukemia; ANA, antinuclear antibody; BSCT, blood stem cell transplantation; cGVHD, chronic graft-versus-host disease; CRP, C-reactive protein; GVHD, graft-versus-host disease; HLA, human leukocyte antigen; HSCT, hematopoietic stem cell transplantation; JIA, juvenile idiopathic arthritis; MCPs, metacarpophalangeal joints; MMP-3, matrix metalloproteinase-3; MTX, methotrexate; N/A, not applicable; NSAIDs, non-steroidal anti-inflammatory drugs; PBSCT, peripheral blood stem cell transplantation; PIPs, proximal interphalangeal joints; PSL, prednisolone; RA, rheumatoid arthritis; RF, rheumatoid factor; SCT, stem cell transplantation; TCZ, tocilizumab.

Citation (Author, Year)	Patient Details (Age in years/Sex)	Primary Disease for HSCT	Type of Transplant	Donor	Time to RA/JIA Onset	Diagnostic Basis	Chimerism (Allogeneic)	Presence of GVHD	Treatment and Outcome
Imamura R, et al. (2002) [3]	51/Male	Non-Hodgkin's Lymphoma	Autologous PBSCT	N/A	40 days	Clinical criteria, RF positive (pre-existing), HLA-DRB1*0405 positive	N/A	No	Prednisolone, diclofenac; Improved
Barnabe CC, et al. (2009) [4]	24/Male	T-cell lymphoma	Autologous PBSCT (CD34-selected)	N/A	~10 months	Symmetrical polyarthritis (PIPs, MCPs, wrists, knees), ANA positive post-SCT	N/A	No	Not reported
Barnabe CC, et al. (2009) [4]	21/Female	AML	Allogeneic SCT	HLA-matched brother	~1 year	Oligoarthritis (shoulders, hip, knee)	Not reported	Yes (acute skin, chronic lungs)	Steroid injection; Improved
Daikeler T, et al. (2011) [5]	Not reported	Systemic Sclerosis	Autologous HSCT	N/A	Not reported	Diagnosed as RA	N/A	No	Methotrexate, abatacept; Ongoing
Daikeler T, et al. (2011) [5]	Not reported	Systemic Sclerosis	Autologous HSCT	N/A	Not reported	Diagnosed as RA	N/A	No	NSAIDs (initially); Ongoing without therapy
Daikeler T, et al. (2013) [1]	Not reported	Not reported	Allogeneic Cord Blood Transplant	Unrelated	Not reported (Cohort median 212 days)	Diagnosed as RA (co-morbid with Evans Syndrome)	Not reported (Cohort 92% full donor)	Not reported	Not reported
Tronconi E, et al. (2014) [2]	3 at HSCT, 14 at onset/Female	ALL	Allogeneic HSCT	Unrelated	~11 years	ILAR criteria, Polyarticular, RF negative, ANA positive, MRI findings	Not reported	Yes (cGVHD-like skin lesions)	Methotrexate; Complete remission
Present case	12/Male	Anaplastic large cell lymphoma	Allogeneic HSCT	HLA-unmatched mother	3 years	ILAR criteria, Polyarticular, RF negative, ANA positive, MRI findings	100% donor	Yes (acute skin and GI)	Ruxolitinib, PSL, MTX, ADA, TCZ; Complete remission

These observations suggest hyperactivity of the adaptive immune system plays a crucial role in post-HSCT arthritis pathogenesis, likely driven by autoantibody-producing donor-derived lymphocytes, which reflects a broader failure of immune tolerance establishment during reconstitution.

The failure of multiple single-cytokine-targeted therapies, specifically ADA and TCZ, in our patient further supports the hypothesis that pathogenesis involves broader immune network dysregulation, possibly reflecting a more complex systemic autoimmunity, given the history of myositis and ANA positivity. JAK inhibitors such as Ruxo block the JAK-STAT signaling pathway, which serves as a central hub for various pro-inflammatory cytokines [6] and for T and B cell activation and differentiation [7]. Therefore, the broad-spectrum inhibition provided by Ruxo effectively suppresses both innate cytokine storm and overactive adaptive immune response, offering superior clinical control where single-target agents fail [8].

Notably, Ruxo maintained efficacy without the secondary inefficacy observed with ADA and TCZ. Although the exact mechanism remains speculative since anti-drug antibodies were not measured, neutralizing antibody development is a well-recognized cause of biologic treatment failure [9]. In contrast, the pharmacological properties of Ruxo as a small molecule inhibitor, which does not induce such antibodies, may offer a theoretical advantage in avoiding secondary inefficacy, potentially contributing to sustained clinical remission [10].

Ruxo was well-tolerated without significant adverse events or lymphoma relapse. However, long-term pediatric safety regarding growth and malignancy remains unestablished [11]. Given limited clinical experience in post-HSCT settings, diligent multifaceted follow-up is essential to monitor for potential late-onset complications [12,13]. Finally, the lack of detailed immunologic or cytokine profiling is a limitation of this report, as it restricts our ability to draw definitive mechanistic conclusions.

## Conclusions

Ruxo appears to be a highly effective therapeutic option for refractory secondary JIA after allo-HSCT. Its ability to suppress broad immune dysregulation - encompassing both cytokine networks and adaptive immune hyperactivity - while potentially avoiding secondary inefficacy represents a theoretical clinical advantage. JAK inhibitors should be considered when bDMARDs prove insufficient in this patient population.
